# Low-Dose Exposure to Ganglioside-Mimicking Bacteria Tolerizes Human Macrophages to Guillain-Barré Syndrome-Associated Antigens

**DOI:** 10.1128/mbio.03852-21

**Published:** 2022-02-01

**Authors:** Robert T. Patry, Lauren Essler, Silke Andresen, Frederick D. Quinn, Christine M. Szymanski

**Affiliations:** a Department of Microbiology, University of Georgia, Athens, Georgia, USA; b Complex Carbohydrate Research Center, University of Georgia, Athens, Georgia, USA; c Department of Infectious Diseases, University of Georgia, Athens, Georgia, USA; Iowa State University

**Keywords:** *Campylobacter jejuni*, Guillain-Barré syndrome, gangliosides, lipooligosaccharides

## Abstract

Early in life, commensal bacteria play a major role in immune development, helping to guide the host response toward harmful stimuli while tolerating harmless antigens to prevent autoimmunity. Guillain-Barré syndrome (GBS) is an autoimmune disease caused by errant immune attack of antibody-bound ganglioside receptors on host nerve cells, resulting in paralysis. Lipooligosaccharides enveloping the prevalent enteric pathogen, Campylobacter jejuni, frequently mimic human gangliosides and can trigger GBS by stimulating the autoimmune response. In low- to middle-income countries, young children are consistently exposed to C. jejuni, and it is not known if this impacts GBS susceptibility later in life. Using a macrophage model, we examined the effect of training these cells with low doses of ganglioside-mimicking bacteria prior to challenge with GBS-associated antigens. This training caused decreased production of proinflammatory cytokines, suggesting tolerance induction. We then screened Campylobacter isolates from 154 infant fecal samples for GM1 ganglioside mimicry, finding that 23.4% of strains from both symptomatic and asymptomatic infants displayed GM1-like structures. Training macrophages with one of these asymptomatic carrier isolates also induced tolerance against GBS-associated antigens, supporting that children can be exposed to the tolerizing antigen early in life. RNA interference of Toll-like receptor 2 (TLR2) and TLR4 suggests that these receptors are not involved in tolerance associated with decreases in tumor necrosis factor (TNF), interleukin-6 (IL-6), or IL-1β levels. The results of this study suggest that exposure to ganglioside-mimicking bacteria early in life occurs naturally and impacts host susceptibility to GBS development.

## INTRODUCTION

Bacteria have been implicated in the development of proper human immune function for decades. The original hygiene hypothesis suggested that as the general population becomes more hygienic and families have fewer children, the incidence of childhood infections should decline and children may not be exposed to bacteria important for preventing allergies (1). Since then, the importance of bacteria in promoting immune homeostasis has been repeatedly supported, being crucial not only in the prevention of allergy but also in several noncommunicable diseases. Humans are colonized in the earliest moments of life, and the bacteria that they are exposed to during this stage have profound and lasting impacts on immune function (2, 3). The composition of a child’s intestinal microbiota is impacted by several factors, including mode of birth (whether vaginal or by caesarean section), exposure to antibiotics, diet and dietary supplements, probiotics, and environmental exposure (4). Healthy conditions influence a complex ecosystem of microbes that help in programming the immune system to tolerate harmless antigens, preventing allergies and autoimmune diseases while responding to markers associated with harmful infection (5). However, unhealthy conditions can lead to a dysbiotic microbiota, which later impacts susceptibility to infection (6) as well as many other conditions, including allergies (7–10), asthma (8, 11, 12), atopic dermatitis (8, 13), type 1 diabetes (14), type 2 diabetes (15), arthritis and other connective tissue diseases (16–18), inflammatory bowel disease (19, 20), immunodeficiencies (21), leukemia (22), obesity (23), and neurological conditions (24).

An important example of how bacteria can induce tolerance by the host immune system is endotoxin tolerance. Endotoxins are lipopolysaccharides (LPS) that extensively comprise the outer membrane of most Gram-negative bacteria. Endotoxin tolerance occurs when immune cells are stimulated with LPS and temporarily show abrogated immune responses to subsequent challenge (25). In mice, the intestinal epithelial cells acquire endotoxin tolerance through exposure to bacterial LPS immediately after birth when delivered vaginally but not by caesarian section (26). Asthma incidence is lower in houses where dust contains increased amounts of LPS, particularly for children raised on farms, where incidence of asthma and allergies is inversely related to LPS levels in the dust, bedding, and mattresses (27, 28). Bashir et al. also reported that Toll-like receptor 4 (TLR4)-deficient mice are more susceptible to food allergy, and a similar effect is observed for normal mice treated with broad-spectrum antibiotics shortly after birth, implicating TLR4 recognition of LPS as the likely cause of this phenomenon (29). It is important to note that the structure of the LPS and antigenicity of its lipid A portion are both important factors in how the immune system recognizes and responds to the antigen. In some cases, these molecules are responsible for the induction of autoimmune disease rather than prevention, as is the case with Campylobacter jejuni-induced Guillain-Barré syndrome (GBS).

In addition to being the most common form of acute paralytic neuropathy, GBS is also the most severe and is frequently associated with antecedent infections (30). Knowledge of this tendency to follow infection was recently reinforced with reports of increased incidence during the Zika virus epidemics in French Polynesia and Latin America (31–33) and now potentially are associated with specific vaccines against SARS-CoV-2 (34, 35). The pathogen most commonly associated with GBS is C. jejuni, with some reports implicating the microbe in up to 39% of cases (36, 37). C. jejuni strains possess lipooligosaccharide (LOS) structures that can mimic various human gangliosides and can induce autoreactive antibody production against ganglioside receptors abundantly found on peripheral motor neuron axons, leading to this autoimmunity following infection (38, 39). It is estimated that 60% of C. jejuni strains produce these mimics (40) and that 1:1,000 C. jejuni infections results in GBS (41, 42). One serotype commonly associated with this disease is HS:19, represented by the type strain we will refer to here as HS:19. This is likely due to its ability to display two different GBS-causing ganglioside mimics on its surface simultaneously (GM1 and GD1a) (43). Previous studies report that patients often produce antibodies that recognize ganglioside complexes in addition to individual gangliosides (44, 45). The ability of HS:19 to display both gangliosides simultaneously allows for production of autoreactive antibodies against both GM1 and GD1a and a combined complex of the two.

C. jejuni is among the primary causes of bacterial diarrhea worldwide, impacting both developed and developing nations. However, in low- to middle-income countries (LMICs), the pathogen is associated with a high incidence of childhood mortality (46, 47). In these nations, the most common sources of C. jejuni are contaminated food and water, and those most susceptible to campylobacteriosis are children under the age of two (48). Importantly, it is also common for these children to be transiently exposed to C. jejuni and shed the bacterium but not experience disease symptoms (48). The Global Enteric Multicenter Study (GEMS) was designed to investigate the causes and impact of diarrheal disease in LMICs for children up to 5 years of age (49). This study collected samples from 4 countries in sub-Saharan Africa (The Gambia, Kenya, Mali, and Mozambique) and 3 in South Asia (Bangladesh, India, and Pakistan) (49). For a previous study, we received a number of Campylobacter strains isolated from infant feces within the GEMS cohort (50).

This study aims to determine if exposure to low levels of ganglioside-mimicking LOS can tolerize immune cells and abrogate their proinflammatory response to subsequent GBS antigen challenge, potentially influencing susceptibility to the disease. Previous studies have described raised levels of proinflammatory cytokines, such as tumor necrosis factor (TNF), interleukin-6 (IL-6), and IL-1β, in GBS patients (51–54) and demonstrated their contribution to the symptoms of the disease (55, 56). With macrophages being key producers of these cytokines, a human macrophage model was employed with C. jejuni HS:19 as the challenge to test the hypothesis. Following these experiments, we analyzed the GEMS infant isolates, determining how many were capable of mimicking GM1 gangliosides and exposing their infant host to this antigen during the stage of life critical for development of immune tolerance.

## RESULTS

### Exposure to GM1 ganglioside mimicking bacteria abrogates proinflammatory responses to Guillain Barré syndrome-causing bacteria in macrophages.

**FIG 1:**
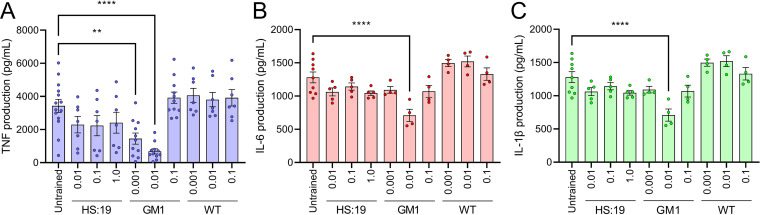
Training with E. coli GM1 induces tolerance to C. jejuni HS:19 challenge. The release of TNF (A), IL-6 (B), and IL-1β (C) after training macrophages with C. jejuni HS:19 (HS:19) and E. coli strain CWG308 wild type (WT) and from the WT engineered to express the GM1 mimic (GM1) at the indicated multiplicities of infection (MOI) and subsequently challenging with C. jejuni HS:19 at an MOI of 5. Each data point represents one biological replicate. Error bars indicate standard errors of the means. ****, *P* < 0.0001; **, *P* ≤ 0.01, determined by one-way ANOVA.

To assess the influence of prior exposure to ganglioside-mimicking bacteria on subsequent challenge with GBS-causing bacteria, the human leukemia monocytic THP-1 cell line was differentiated into macrophages in the presence or absence of paraformaldehyde-treated ganglioside-mimicking bacteria at low multiplicity of infection (MOI). After this training stage, the macrophages were challenged with high doses of paraformaldehyde-treated ganglioside-mimicking bacteria known to be capable of causing GBS (HS:19 at an MOI of 5). The bacteria used in training were either HS:19 itself, E. coli CWG308 pCst/pGM1a (E. coli GM1) that was engineered to display the same mimics on a truncated lipopolysaccharide (LPS), or E. coli CWG308 wild type (E. coli WT), the parent strain of the engineered E. coli, displaying a truncated LPS with no mimic present (Fig. 1) (57). After challenge, the strength of the inflammatory immune response was determined by measuring the concentration of several proinflammatory cytokines produced in the supernatant of the macrophage cell culture.

These training experiments showed that exposure to low MOIs of HS:19 or E. coli GM1 resulted in a downward trend in TNF production for both HS:19 and E. coli GM1 over several MOIs; however, statistical significance was only achieved when E. coli GM1 was used for training at an MOI of 0.001 (*P = *0.0016) or 0.01 (*P < *0.0001) (Fig. 1A). When E. coli GM1 was used at a higher MOI, the release of TNF returned to untrained levels (Fig. 1A). Further analysis using the same training conditions with no subsequent HS:19 challenge revealed that this was due to the ganglioside-mimicking E. coli now inducing TNF production in the absence of a challenge stimulus (see Fig. S1A in the supplemental material). No trends were observed when macrophages were trained with the E. coli WT parent strain lacking the ganglioside mimic. IL-6 and IL-1β were also measured in subsequent experiments and showed results similar to those seen for TNF (Fig. 1B and C). For IL-6, the downward trend was noticeable when cells were trained with HS:19 and significantly decreased using E. coli GM1 at an MOI of 0.01 (*P = *0.0210) (Fig. 1B). As was the case with TNF, E. coli GM1 induced IL-6 secretion at an MOI of 0.1 (Fig. 1B) regardless of the addition of HS:19 challenge (Fig. S1B). Training with E. coli WT did not result in decreased IL-6 release (Fig. 1B). Training with HS:19 and E. coli GM1 caused a decline in IL-1β, with statistical significance for E. coli GM1 at an MOI of 0.01 (*P < *0.0001) and no trend seen for E. coli WT (Fig. 1C). Taken together, these results show that training of macrophages with a low MOI of GM1 ganglioside-mimicking bacteria can cause a decrease in the magnitude of proinflammatory responses directed at that same antigen in subsequent exposures. It also suggests that this effect is even more pronounced when training with E. coli GM1 than with a homologous challenge.

10.1128/mbio.03852-21.1 FIG S1 Cytokine release after training macrophages without challenge. Download FIG S1, PDF file, 0.2 MB. Copyright © 2022 Patry et al. 2022 Patry et al. https://creativecommons.org/licenses/by/4.0/ This content is distributed under the terms of the Creative Commons Attribution 4.0 International license.

### Lipooligosaccharides from ganglioside-mimicking bacteria are sufficient to reduce the TNF response against Guillain Barré syndrome-causing bacteria.

To further support that the primary structure involved in bacterium-tolerizing macrophages to GBS antigens is their LOS, the LOS of HS:19, E. coli GM1, and E. coli WT was isolated and used as the training stimulus, replacing ganglioside-mimicking whole bacteria in the THP-1 model to induce tolerance prior to HS:19 challenge. As an additional control to differentiate these results from what could be attributed to routine LPS tolerance, a commercial E. coli LPS was added. The concentrations of LPS and LOS were determined using those described in THP-1 cell literature (58) in addition to our own pilot experiments (data not shown).

**FIG 2:**
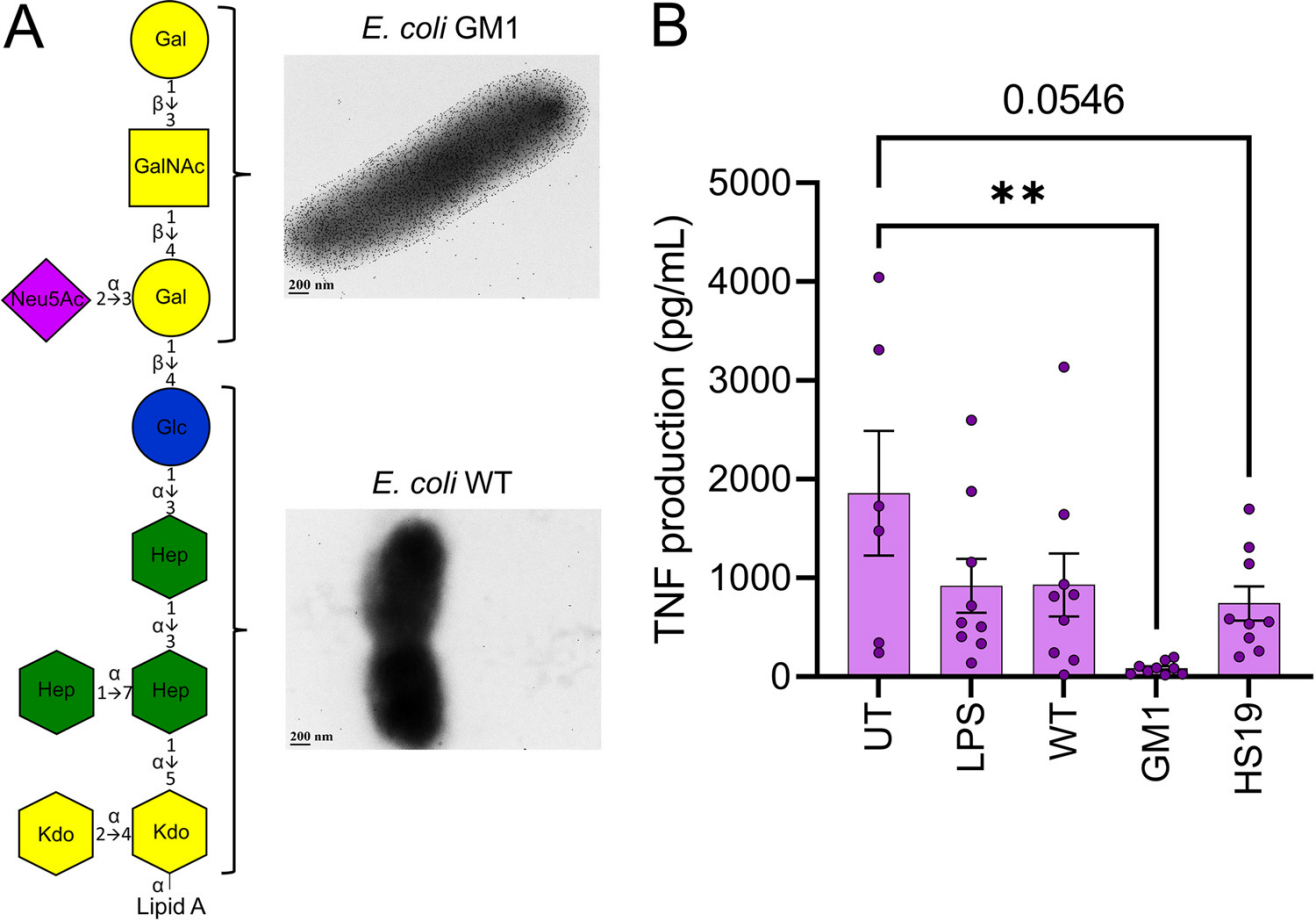
Training with purified lipooligosaccharides (LOS) from E. coli GM1 is sufficient to induce tolerance to C. jejuni HS:19 challenge. (A) Structures of LOS from E. coli strain CWG308 wild type (WT) and from the WT engineered to express the GM1 mimic (GM1) are shown using the Consortium for Functional Glycomics symbol nomenclature (100). Confirmation of GM1-mimic expression was described in Focareta et al. (57) and shown here by binding with immunogold-labeled cholera toxin B subunit followed by transmission electron microscopy. Scale bars are indicated in each panel. (B) Macrophages were trained with commercial lipopolysaccharide (LPS) or purified LOS from C. jejuni HS:19 (HS:19), E. coli GM1 (GM1), or E. coli WT (WT) before challenging with C. jejuni HS:19 at a multiplicity of infection of 5. Each data point represents one biological replicate. Error bars represent the standard errors of the means. **, *P* ≤ 0.01 determined by one-way ANOVA.

When macrophages were trained with either the purchased LPS or LOS isolated from E. coli WT, there was a downward trend observed in TNF production; however, this difference was not statistically significant (*P* = 0.1262 and 0.1309, respectively) (Fig. 2). Training with the LOS of HS:19 resulted in a further decrease, approaching statistical significance (*P* = 0.0546) (Fig. 2). As expected from the previous results, the most dramatic decrease in TNF release was observed by training with E. coli GM1 LOS (*P* = 0.0012) (Fig. 2). Furthermore, the number of nonviable cells was similar between untrained cells and those trained with LOS from C. jejuni HS:19 or with E. coli GM1 LOS (Fig. S1B). These data support that the ganglioside-mimicking portion of these tolerizing bacteria (their LOS) is not only necessary to dampen the release of proinflammatory cytokines against GBS-causing organisms but also sufficient.

### Tolerance by ganglioside-mimicking bacteria is not mediated through TLR2 or TLR4.

**FIG 3:**
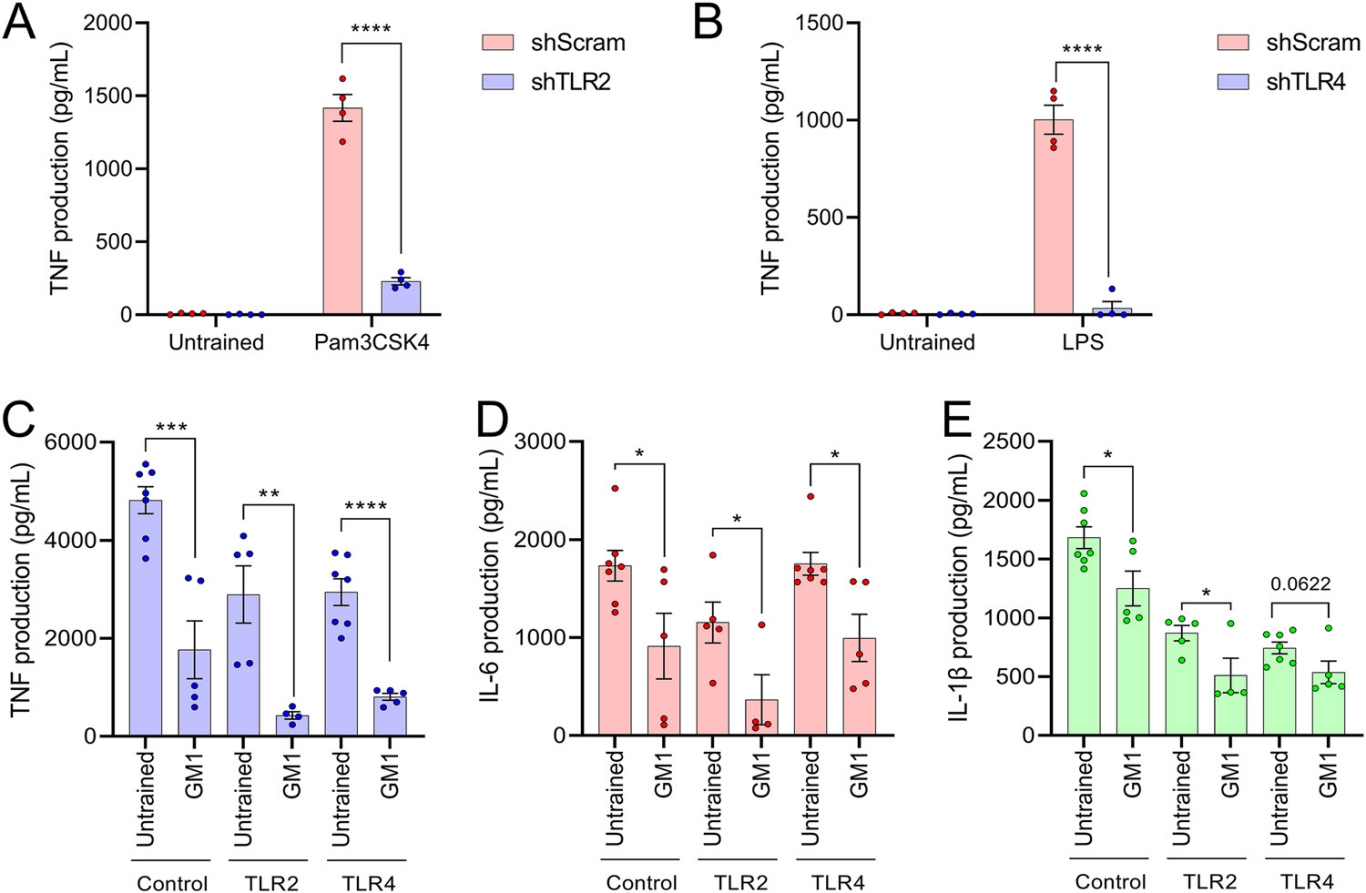
Neither TLR2 nor TLR4 is responsible for lipooligosaccharide-induced GM1-ganglioside tolerance. Confirmation of TLR2 knockdown (shTLR2) (A) and TLR4 knockdown (shTLR4) (B) macrophages using known agonists for the TLRs including Pam3CSK4 (for TLR2) or commercial lipopolysaccharide (LPS; for TLR4) compared to short hairpin loop RNA control (shScram) macrophages. The release of TNF (C), IL-6 (D), and IL-1β (E) after training shScram, shTLR2, or shTLR4 macrophages with C. jejuni HS:19 (HS:19), E. coli GM1 (GM1), or E. coli WT (WT) at a multiplicity of infection (MOI) of 0.01 and subsequently challenging with C. jejuni HS:19 at an MOI of 5. Each data point represents one biological replicate. Error bars represent standard errors of the means. ****, *P* < 0.0001; ***, *P* ≤ 0.001; **, *P* ≤ 0.01; *, *P* ≤ 0.05, determined by the Student's *t* test.

Once it was discovered that the LOS of GM1-ganglioside-mimicking bacteria can tolerize macrophages to challenge with HS:19, the next step was to determine the mechanism for this decrease in inflammatory response. To test this, TLR2 and TLR4 in the macrophages were silenced using gene-specific lentiviral short hairpin RNA (shRNA) and compared to nonspecific scrambled lentiviral shRNA as a negative control. The knockdowns were validated using flow cytometry (Fig. S3), and their impaired ability to signal production of TNF was tested using known ligands Pam3CSK4 for TLR2 (Fig. 3A) and E. coli LPS for TLR4 (Fig. 3B). For the training experiments, E. coli GM1 was used to induce tolerance at an MOI of 0.01, since this training treatment consistently resulted in the most robust tolerance to GBS antigen.

10.1128/mbio.03852-21.2 FIG S2 Viability staining of THP-1 cells before and after training. Download FIG S2, PDF file, 0.5 MB. Copyright © 2022 Patry et al. 2022 Patry et al. https://creativecommons.org/licenses/by/4.0/ This content is distributed under the terms of the Creative Commons Attribution 4.0 International license.

10.1128/mbio.03852-21.3 FIG S3 Flow cytometry data from TLR knockdown cell lines. Download FIG S3, PDF file, 0.3 MB. Copyright © 2022 Patry et al. 2022 Patry et al. https://creativecommons.org/licenses/by/4.0/ This content is distributed under the terms of the Creative Commons Attribution 4.0 International license.

When trained with E. coli GM1 and challenged with HS:19, macrophages treated with scrambled shRNA (control) again displayed a tolerance trend following E. coli GM1 training compared to cells left without training, as expected (Fig. 3C to E). This was the case with TNF (*P=* 0.0004), IL-6 (*P=* 0.0342), and IL-1β (*P=* 0.0257). For the macrophages treated with TLR2-specific shRNA (TLR2), tolerance was again shown, replicating what was seen in the control for TNF (*P* = 0.0075), IL-6 (*P = *0.0458), and IL-1β (*P = *0.0471). Finally, for cells treated with shRNA specific to TLR4 (TLR4), the results for TNF (*P < *0.0001) and IL-6 (*P = *0.0113) were also similar to those for the control cells, with tolerance in macrophages trained with E. coli GM1. There was also a decrease observed in IL-1β following E. coli GM1 training (Fig. 3E); however, this result was not statistically significant (*P = *0.0622). It is interesting that production of all three cytokines in the TLR2 macrophages was lower than what was observed in the control cells. Since the tolerance effect was not changed by silencing either TLR2 or TLR4, it is not likely that either of these receptors is the driving factor of the phenomenon.

### Children in low- and middle-income countries are exposed to ganglioside-mimicking bacteria early in life.

**FIG 4:**
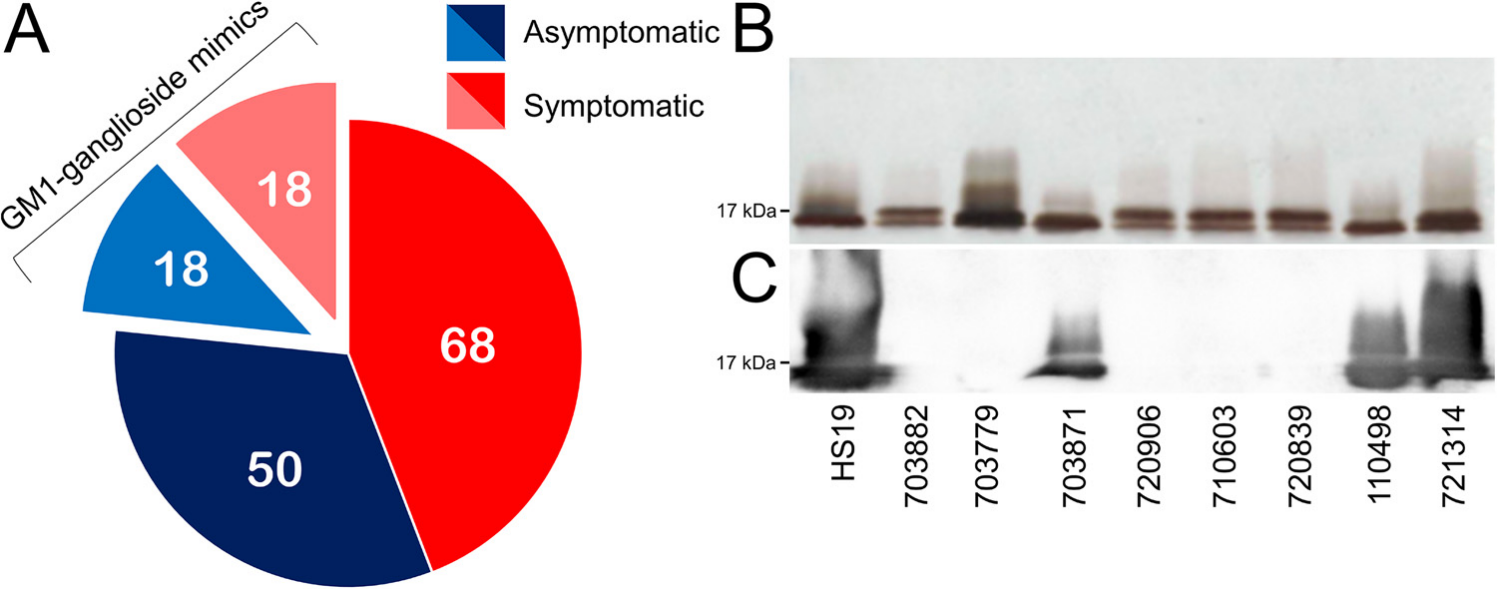
Infants in low- and middle-income countries are exposed to GM1 ganglioside-mimicking C. jejuni. (A) A pie chart of infant Campylobacter isolates separated by display of symptoms (asymptomatic are blue, symptomatic are red) and highlighting the number of GM1 ganglioside mimic-positive samples within each group. (B) Example silver stain of lipooligosaccharides from Campylobacter isolates and (C) the corresponding Western blot using cholera toxin B subunit as a probe. C. jejuni HS:19 was used as a positive control. Unedited silver stain and Western blot images can be found in Fig. S3.

A GM1 ganglioside mimic screen was done comparing isolates collected from 154 fecal samples from symptomatic (86) or asymptomatic (68) Campylobacter-infected infants under 1 year of age from the GEMS study (49). More information regarding each sample collected from 7 different nations is listed in Table S1. LOS was isolated from each sample (confirmed by silver stain) (Fig. 4A) and tested for GM1 ganglioside mimicry using cholera toxin B subunit (CTB) as the antigen probe (Fig. 4B). HS:19 was used as a positive control. In total, 36/154 isolates (23.4%) were positive for GM1 mimics, and, of these, half came from symptomatic and half were from asymptomatic infants (Fig. 4C). GM1-positive isolates originated from all nations involved in the study, indicating their presence was not concentrated at one particular site (Table S3). These data show that individuals can be exposed to ganglioside-mimicking bacteria early in life and that their presence may not be accompanied by disease symptoms from an inflammatory response.

10.1128/mbio.03852-21.5 TABLE S1 Description of infant fecal isolate samples. Download Table S1, PDF file, 0.1 MB. Copyright © 2022 Patry et al. 2022 Patry et al. https://creativecommons.org/licenses/by/4.0/ This content is distributed under the terms of the Creative Commons Attribution 4.0 International license.

### Ganglioside-mimicking bacteria isolated from low- and middle-income infants can tolerize against GBS-causing antigens.

**FIG 5:**
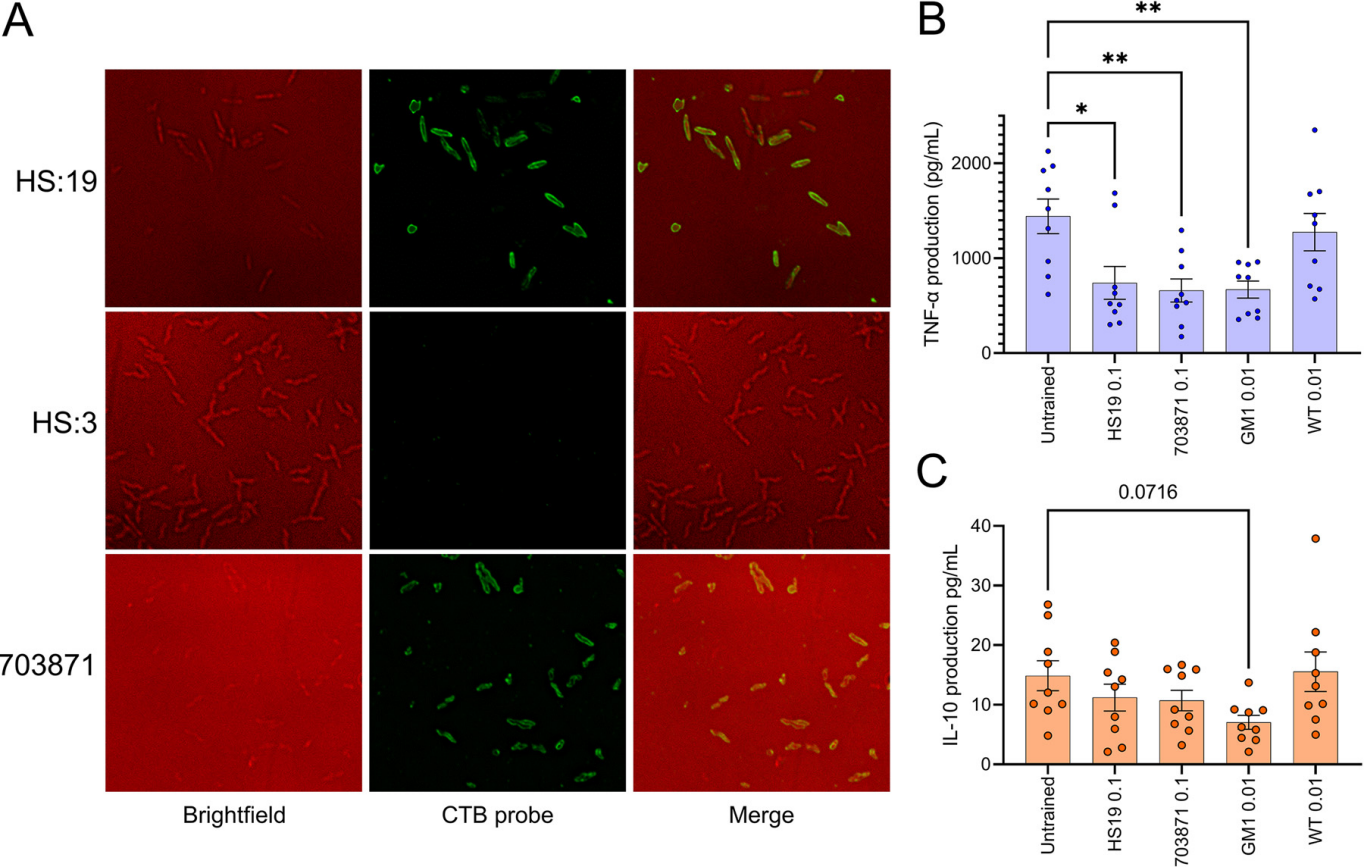
Training with C. jejuni strain 703871 also induces tolerance to C. jejuni HS:19 challenge, which is not mediated by IL-10 release. (A) Fluorescence microscopy done on a GM1-positive infant isolate (703871) using a CTB probe with C. jejuni HS:19 as a positive control and C. jejuni HS:3 as a negative control along with graphs depicting the release of TNF (B) or IL-10 (C) after training macrophages with C. jejuni HS:19 (HS:19), E. coli GM1 (GM1), and E. coli WT (WT) at their respective optimal tolerizing multiplicities of infection (MOI; 0.1 or 0.01) and subsequently challenged with C. jejuni HS:19 at an MOI of 5. Each data point represents one biological replicate. Error bars represent the standard errors of the means. **, *P* ≤ 0.01; *, *P* ≤ 0.05 determined by one-way ANOVA test.

Following the observation that infants from low- and middle-income countries can harbor ganglioside-mimicking Campylobacter, we wanted to determine if the presence of those bacteria could tolerize macrophages against GBS-causing antigens in the same manner as that observed for the previous experiments. To pursue this question, an isolate was selected that had LOS that bound to CTB and was from an infant without symptomatic campylobacteriosis. Isolate 703871 (Table S1) was selected after confirming homogeneous production of GM1-mimicking LOS from individual cells by fluorescence microscopy (Fig. 5A). This confirmation was important to establish due to the tendency of C. jejuni LOS structures, particularly ganglioside mimics, to be phase variable (59, 60). When trained with HS:19 (*P = *0.0114) or E. coli GM1 (*P = *0.0049) prior to challenge with HS:19 as done before, tolerance once again was observed as TNF production was significantly reduced compared to that of untrained macrophages. This was not the case for the E. coli WT strain. In addition, training with the isolated C. jejuni 703871 showed a significant reduction in TNF release (*P = *0.0043). This result supports that in areas where ganglioside-mimicking bacteria are endemic, people are exposed to GBS antigens from an early age, potentially having a tolerogenic influence on responses against gangliosides that lead to GBS.

### IL-10 is not stimulated in response to exposure to ganglioside-mimicking bacteria.

At this point in the study, we focused on tolerance due to the suppression of proinflammatory cytokines but did not explore the possibility that exposure to ganglioside-mimicking bacteria could influence anti-inflammatory cytokine release as well. Endotoxin-tolerant macrophages are often thought to produce less proinflammatory cytokines such as TNF and more anti-inflammatory cytokines such as IL-10 (61). To investigate the potential role of IL-10 in our model, its release was measured following exposure to ganglioside-mimicking bacteria in the same THP-1 macrophage model as that used for previous experiments. No significant differences in IL-10 levels were observed between untrained cells and those trained with any of the bacteria from the study; however, there was a decrease associated with E. coli GM1 training that was nearly significant (*P* = 0.0716). These results suggest that IL-10 is not involved in the tolerance response that we have observed and is potentially suppressed by training with ganglioside mimics in a way similar to that of the proinflammatory cytokines.

## DISCUSSION

Although extensive progress has been made in determining the cause of GBS sequelae and the role infectious agents play in precipitating this autoimmune disorder, very little is still known about host factors that impact GBS susceptibility in individuals. In allergy (7–10), asthma (8, 11, 12), diabetes (14, 15), atopic dermatitis (8, 13), arthritis (16–18), and other autoimmune-related diseases, the commensal microbiota plays a key role in promoting tolerance to self-antigens early in life to help shape the immune system and prevent aberrant reactions later in life. Our study began by developing an assay to examine the role of ganglioside-mimicking bacteria in the prevention of proinflammatory reactions to GBS-causing antigens. Human THP-1 monocyte cells can be treated with phorbol 12-myristate-13-acetate (PMA) to induce differentiation into macrophages and is a widely used model to investigate cellular immunity. After differentiation with PMA, the macrophages can then polarize to display either M1 or M2 phenotypes depending on the stimuli they are exposed to. M1 macrophages polarized by LPS are proinflammatory in nature, producing cytokines such as TNF, IL-6, IL-12, IL-23, and IL-1β, while M2 macrophages are polarized by a number of different stimuli and can assume several distinct phenotypes (M2a, M2b, M2c, and M2d) (62). TLR ligands tend to stimulate the M2b and M2d types, both producing high levels of IL-10, but M2b also secretes TNF, IL-6, and IL-1β (62). TNF released by macrophages plays a role in the development of GBS symptoms, and exposure to anti-TNF improves the outcomes (63). Additionally, injecting TNF or IL-6 into nerve tissue worsens clinical signs (64). Our experiments utilizing THP-1 cells suggest that when macrophages are exposed to low doses of ganglioside-mimicking bacteria during differentiation, they become tolerized to the ganglioside antigen, and proinflammatory cytokine release is reduced upon subsequent exposure to GBS-associated bacteria. However, there was no observed change in release of the anti-inflammatory cytokine IL-10. This result is not surprising; while some previous studies have shown an increase in IL-10 production by endotoxin-tolerant macrophages (65, 66), others have shown a decrease (67, 68) or evidence that IL-10 is not involved in endotoxin tolerance at all (69). In addition to C. jejuni HS:19, which is known to initiate GBS, the pair of isogenic E. coli GM1 and E. coli WT strains were used because they differ only in their LOS outer core structures. E. coli GM1 displays a terminal GM1 ganglioside mimic comprised of Gal-β-1,3-GalNAc-β-1,4-[Neu5Ac-α-2,3]-Gal while E. coli WT does not (57). Using these strains, we examined the impact of ganglioside mimicking LOS while properly accounting for other antigens on the cell surface. The importance of the LOS ganglioside mimic alone in the tolerogenic mechanism was further exemplified when the structure was isolated from each of the microbes and shown to still induce tolerance to the GBS antigen. Interestingly, our data suggest that E. coli GM1 whole bacterium or LOS alone induces tolerance against C. jejuni HS:19 more effectively than homologous challenge with the HS:19 bacterium or its LOS. It was previously shown that the immunogenicity of LPS structures (i.e., LOS with additional O-antigen repeats) has a distinct impact on their ability to induce innate immune signaling and endotoxin tolerance (70). The more potent of an innate immune activator, the more capable a particular LPS is of endotoxin tolerance induction (70). In C. jejuni, the dehydrogenase GnnA and transaminase GnnB allow for lipid A modification by mediating the replacement of an ester-linked acyl chain with an amide-linked chain (71). This showed increased resistance to various antimicrobial peptides but also reduced endotoxin activity and avoided activation of innate host defenses mediated through TLR4 compared to the E. coli lipid A (71). The difference in immune recognition between these two molecules is further supported by the higher proinflammatory cytokine secretion observed with E. coli GM1 even when the cells were not challenged with HS:19, especially at the higher MOI of 0.1. This increase in lipid A immunogenicity is why E. coli was used at a lower MOI than C. jejuni HS:19 and may explain why E. coli GM1 was better able to induce tolerance than HS:19. The success in developing tolerance using this model supports that exposure of individuals to low levels of ganglioside-mimicking bacteria may have a protective effect against future GBS development.

In LMICs, Campylobacter species are extensively infecting infants and young children during key stages of immune development (49, 50). From an autoimmune disease perspective, this is significant because C. jejuni is the pathogen most commonly associated with eliciting GBS by mimicking human ganglioside structures. Given the propensity of C. jejuni isolates to display ganglioside mimics and high probability of infants in LMICs to be exposed to these organisms, we hypothesized that infants in LMICs are exposed to ganglioside-mimicking Campylobacter strains that could later impact their susceptibility to GBS. Our screen using CTB as a probe for GM1 ganglioside structures confirmed that these infants can be colonized with ganglioside-mimicking Campylobacter strains for an undetermined period. Interestingly, half of the mimicking isolates that we observed were from infants that did not show any signs of campylobacteriosis, suggesting that the presence of the mimic did not influence gastroenteritis and that many children are exposed to these ganglioside-mimicking strains without taking notice. Armed with the knowledge that people can be exposed to ganglioside mimics during infancy, we then sought to study whether the presence of these bacteria impacts the immune system’s tolerance of these antigens upon future exposure. It has been shown in multiple studies that Campylobacter infection leads to increased intestinal and systemic inflammation, which is associated with growth stunting in children (72). Several studies have demonstrated that the granulocyte enzyme myeloperoxidase can be used as a quantitative marker to measure the intestinal inflammation accompanying infection (73–75). A recent investigation, named the Malnutrition and Enteric Disease Study (MAL-ED), examined 26,267 diarrheal and 7,601 nondiarrheal stool samples from 1,892 children across Brazil, Peru, South Africa, Tanzania, India, Pakistan, Bangladesh, and Nepal (75). In this study, various fecal markers of intestinal inflammation were detected, including neopterin (NEO), which can be used to estimate T-helper cell 1-activated cellular immunity (75). Their results showed that the NEO concentrations were actually lower during Campylobacter infection, hinting that the inflammation caused by Campylobacter is driven by innate rather than adaptive immune responses (75). This makes our newly developed macrophage model highly useful for further analysis of the Campylobacter isolates that were provided from the GEMS (49).

C. jejuni isolate 703871 was chosen for further analysis because it showed constitutive display of GM1 mimics and was obtained from an asymptomatic infant. When used to train macrophages against challenge with C. jejuni HS:19, strain 703871 was also able to induce tolerance to the GBS antigen, protecting against the proinflammatory response. It is important to note that we are not suggesting that the presence of Campylobacter early in life is healthy; in contrast, their presence is often accompanied by malnutrition and intestinal inflammation, which can lead to growth stunting (73–76). However, the presence of ganglioside-mimicking bacteria capable of tolerizing against GBS-associated C. jejuni in a healthy infant supports the notion that bacteria present in the human gut, particularly during critical times of immune development, could be one factor in distinguishing GBS susceptibility between individuals. We recently discovered that the chicken intestinal tract is a rich source of ganglioside-mimicking enterococci, in addition to C. jejuni (60), which presents a plausible route of entry for these organisms through the diet and suggests that they are a natural member of other host microbiota. This provides the opportunity to investigate new therapeutics directed at reducing individual susceptibility to GBS by optimizing commensal microbiota composition early in life. However, to follow this path, more needs to be known about the mechanism involved in the tolerance observed and the receptors involved.

Tolerance induced by LPS and LOS results from changes in TLR signaling and primarily involves TLR4 (77). Cytokine production stimulated by LPS can also be reduced by cross-tolerance signaling when TLR2 is exposed to alternate ligands such as lipopeptides (78), lipoarabinomannans (79), soluble tuberculosis factor (79), lipoteichoic acids (80), and zymosan (81, 82). These two TLR receptors are particularly important in regulating inflammatory responses generated in the gut and are both expressed on gut epithelial cells and immune cells of the lamina propria, such as macrophages (83). Upon binding, TLR2 and TLR4 both recruit MyD88, leading to activation of transcription factors such as NF-κB and production of proinflammatory (e.g., TNF, IL-6, and IL-1β) or anti-inflammatory (e.g., IL-10) cytokines (83). In particular, TLR4 exists in a delicate balance between proinflammatory and anti-inflammatory signaling through its differential expression on the cell surface and intracellularly, respectively (84). C. jejuni has been shown to interact with the innate immune system via TLR2 (85) and TLR4 (86), among other receptors (87). Although researchers have reported LPS signaling through TLR2 (88–90), TLR4 is the receptor most commonly associated with LPS (91, 92). Both receptors can induce and suppress the production of proinflammatory cytokines depending on the antigen they bind and the quantity they encounter (77, 92). Therefore, we targeted TLR2 and TLR4 for knockdown using shRNA to determine if either of these receptors was responsible for the tolerance signaling that we observed. After silencing these receptors and testing with the same macrophage model by training with E. coli GM1 or WT and challenging with C. jejuni HS:19, our results suggest that silencing of TLR2 or TLR4 did not impact the decrease in TNF, IL-6, or IL-1β secretion associated with E. coli GM1 training. Given that these receptors are not likely responsible for the tolerance mechanism that we have observed, we are now using both targeted and unbiased approaches to determine the correct receptors involved. A plausible target for this investigation would be the sialic acid-binding immunoglobulin type lectins, referred to as Siglecs, which specifically bind to structures containing sialic acids such as gangliosides. Although there have been differing reports on which Siglecs recognize C. jejuni ganglioside-mimicking LOS, the most recent studies report that Siglec 1 (also known as sialoadhesin or CD169) (93) and Siglec 7 (94, 95) recognize C. jejuni GM1-LOS and are expressed by both monocytes and monocyte-derived macrophages. Future studies will assess the contribution of Siglecs through RNA interference (RNAi) knockdowns and transcriptome sequencing (RNA-seq) experiments.

In conclusion, this study has determined that infants in LMICs are exposed to ganglioside-mimicking C. jejuni early in life and that this does not always involve symptomatic infections that are treated. We also showed that GM1-mimicking LOS displayed on the bacterial surface can induce innate immune tolerance to subsequent challenge with high doses of GBS-associated C. jejuni serotypes. Although more experiments are needed to investigate the mechanism for this tolerance, the differences we observed indicate that ganglioside-mimicking bacteria present in the gut during immune development impact an individual’s susceptibility to GBS. In addition, further experiments *in vivo* in GBS mouse models are needed to show that the presence of these bacteria correlates with improved disease outcomes. The E. coli strain used in this study was initially intended for use as a probiotic to bind to CT in the gut and treat/prevent cholera (57). The knowledge that this strain can also induce tolerance to GBS-associated antigens suggests that in the future, probiotics could serve preventative roles to defend against GBS development.

## MATERIALS AND METHODS

### Bacterial growth conditions.

Campylobacter fecal isolates were streaked from frozen onto Campy-Line agar (CLA) (96) and grown overnight at 37°C under microaerobic conditions. Resulting growth was then restreaked onto brain heart infusion (BHI) agar and incubated as before to use in experiments. E. coli cells were streaked from frozen onto LB agar and grown overnight at 37°C. Colonies from the resulting plates were used to inoculate overnight cultures in liquid LB broth and grown overnight at 37°C with agitation. E. coli GM1 was grown in medium supplemented with ampicillin (50 μg/ml) and kanamycin (25 μg/ml). C. jejuni wild-type cultures were streaked from frozen onto NZCYM agar and grown overnight at 37°C under microaerobic conditions. The resulting colonies were restreaked onto another NZCYM plate and grown as before to use in experiments.

### Preparation of paraformaldehyde-treated bacterial cells.

After culturing, C. jejuni and E. coli were resuspended and washed in sterile phosphate-buffered saline (PBS). Next, the cells were resuspended in PBS with 4% paraformaldehyde and incubated at room temperature for 30 min. The suspension was then washed three times with sterile PBS. The killed bacteria were counted using a Petroff-Hausser counter and either used directly in monocyte assays or stored at 4°C.

### THP-1 human monocyte-like cell culturing.

The human leukemia monocytic THP-1 cell line was obtained from the American Type Culture Collection (TIB-202) and maintained in RPMI with 2-mM l-glutamine, 1 mM sodium pyruvate, and 10% fetal bovine serum (C-RPMI) at 3 × 10^5^ to 8 × 10^5^ cells/ml.

### THP-1 human monocyte-like cell cytokine release assays.

Experiments in THP-1 cells were based on methods previously described and expanded upon (97). After growth to approximately 8 × 10^5^ cells/ml in C-RPMI, monocyte-like THP-1 cells were pelleted by centrifugation at 200 × *g* for 5 min and resuspended in C-RPMI to a concentration of 1 × 10^6^ cells/ml. PMA was then added to a final concentration of 50 nM, and the cells were seeded in 48-well tissue culture plates at 4 × 10^5^ cells/well. Commercial LPS (LPS-EB from E. coli O111:B4; InvivoGen) at 10 ng/ml, purified LOS at 10 ng/ml, or paraformaldehyde-treated bacteria at different multiplicities of infection (MOI) were added to the wells during this time for training. The cells were allowed to differentiate for 24 h into a macrophage-like phenotype before the medium was discarded and replaced with fresh C-RPMI, and they were rested for 16 h before challenge with C. jejuni HS:19 at an MOI of 5. After 24 h of challenge, the supernatants were removed and cytokine production measured using TNF (BD-Biosciences), IL-6 (BD-Biosciences), or IL-1β (Invitrogen) enzyme-linked immunosorbent assay (ELISA) kits. Cytokine production was measured with technical duplicates in each of several biological replicates.

### Silencing of TLR2 and TLR4 by lentiviral particle transduction.

The TLR2 and TLR4 knockdown cell lines were created using gene-silencing short-hairpin RNA (shRNA) lentiviral particles (Santa Cruz Biotech). Next, 2 × 10^4^ THP-1 cells were transferred to microcentrifuge tubes in C-RPMI with 5 μg/ml Polybrene and TLR2-specific, TLR4-specific, or scramble control lentiviral particles at an MOI of 10. The cells were centrifuged at 900 × *g* for 30 min and then transferred to flat-bottom 96-well plates and incubated overnight at 37°C in 5% CO_2_. Cells were then resuspended in fresh C-RPMI and expanded to 48-well plates. Monolayers were monitored for viability, and stably transduced cells were selected using 1 μg/ml puromycin and then expanded to make liquid nitrogen stocks. Before use in cytokine release assays, thawed cells were passaged once with puromycin.

### Immunogold transmission electron microscopy of E. coli.

The immunogold protocol was described in our previous study (60). Briefly, E. coli WT and GM1 were grown as described above, and the optical density at 600 nm (OD_600_) was measured and adjusted to 1.0. Next, 2 ml of the cells was incubated with 2 μl of CTB (1 mg ml^−1^; Sigma) for 1 h before being washed and resuspended in PBS. The cell suspension was spotted onto Parafilm and a Formvar-coated copper grid laid atop for 1 h. The grid was then blocked with PBS plus 5% bovine serum albumin (BSA) for 1 h and treated with rabbit α-CT antibodies (Fitzgerald Industries International) in PBS plus 5% BSA for 1 h and then goat α-rabbit IgG conjugated to 10-nm gold particles (BB International) in PBS plus 5% BSA. Both antibodies were used at a 1:50 dilution, and grids were washed in PBS plus 5% BSA three times between each step. The grids were imaged using a Philips Morgagni 268 transmission electron microscope (FEI Company) along with a charge-coupled camera and controller (Gatan). The images were processed using DigitalMicrograph (Gatan).

### Preparation of LOS from infant fecal isolates.

LOS preparation was done as described previously, with minor modifications (98). Briefly, isolate growth was harvested into sterile PBS and adjusted to an OD_600_ of 0.375. Next, 1.5 ml was transferred to a new tube and centrifuged for 4 min at 6,200 × *g*. The resulting pellet was resuspended in 150 μl of lysing buffer (100 mM Tris-Cl [pH 8.0], 2% β-mercaptoethanol, 4% SDS, 0.2% bromophenol blue, 0.2% xylene cyanol, 20% glycerol). This mixture was boiled for 10 min at 95°C before cooling to room temperature and adding another 150 μl of lysing buffer and proteinase K to a final concentration of 0.5 mg/ml. The samples were then incubated overnight at 37°C before heating to 65°C for 1 h. These samples were directly loaded onto an SDS-PAGE gel or stored at −20°C.

### Hot phenol preparation of LOS.

Overnight E. coli cultures were used to inoculate 500 ml of LB medium and grown under previously mentioned conditions. C. jejuni HS:19 was cultured and expanded on NZCYM agar, harvested in PBS, and pelleted at 7,649 × *g* until the resulting pellet resembled those obtained from E. coli cultures in quantity. LOS was then isolated using the hot water-phenol extraction method as previously described (99). The resulting pellet was lyophilized in a preweighed tube and then resuspended and analyzed for purity by silver staining and ability to bind CTB by Western blotting using methods previously described (60). The quantity of purified LOS was normalized by measuring the dry weight.

### Far-Western blots of isolate and hot phenol-purified LOS.

Far-Western blots were performed as described previously, with minor modifications (60). Briefly, samples were separated using SDS-PAGE and the resulting gel was wet transferred to a nitrocellulose membrane. Following this, the membrane was blocked in BS (PBST plus 5% skim milk) overnight at 4°C. The membrane was then probed with CTB (1 mg/ml; diluted 1:100,000 in BS; Sigma) for 1 h, rinsed 3 times, and washed 3 times for 5 min in PBST. It was then probed with rabbit α-CT antibodies (1:6,500) for 1 h, washed as before, probed with goat α-rabbit-horseradish peroxidase antibodies (1:20,000) for 1 h, and washed again as before. The membranes were developed using Clarity Western ECL substrate (Bio-Rad), and images were captured using the ChemiDoc XRS system (Bio-Rad).

### Fluorescent microscopy of infant fecal isolates.

After growth as described above, the isolates were harvested from plates in PBS and their OD_600_ was adjusted to 0.05. Next, 2 ml of this suspension was mixed with 2 μl of CTB and incubated for 1 h at room temperature with agitation. The mixture was then centrifuged for 4 min at 6,200 × *g* and washed with PBS 3 times. Next, 10 μl of the mixture was spotted onto a coverslip and left to air dry before heat fixing. The coverslips were blocked for 1 h in blocking solution before being probed with rabbit α-CT antibodies (1:6,500 in BS) for 1 h. They were then washed with PBS 3 times for 5 min and probed with goat α-rabbit-Alexa 488 antibodies (1:500 in BS; Invitrogen) before being washed again as before. The coverslips were rinsed in Milli-Q water and allowed to dry before mounting with VectaShield antifade mounting medium (Vector Laboratories). Samples were imaged using an Olympus IX-71 inverted microscope with a CoolSnap HQ2 camera.

### Statistics.

Statistics presented for THP-1 experiments were done by one-way analysis of variance (ANOVA) with the exception of the shRNA TLR knockdown experiments, where comparisons were done by two-tailed, unpaired *t* test. Results were considered significant when the *P *value was <0.05.

10.1128/mbio.03852-21.4 FIG S4 Full images of the silver stain and Western blot shown in Fig. 4. Download FIG S4, PDF file, 0.1 MB. Copyright © 2022 Patry et al. 2022 Patry et al. https://creativecommons.org/licenses/by/4.0/ This content is distributed under the terms of the Creative Commons Attribution 4.0 International license.
