# Supplementary material for: Low-Dose Exposure to Ganglioside-Mimicking Bacteria Tolerizes Human Macrophages to Guillain-Barré Syndrome-Associated Antigens
Source: mBio. 2022 Feb 1;13(1):e03852-21. doi: 10.1128/mbio.03852-21 (PMC8805021; doi:10.1128/mbio.03852-21)
Supplement: FIG S1 [file mbio.03852-21-sf001.pdf]

Supplemental material – Figure 1

Low-dose exposure to ganglioside-mimicking bacteria tolerizes human macrophages to Guillain-Barré Syndrome-associated antigens

Robert T. Patry[a,b*], Lauren Essler[c], Silke Andresen[a,b], Fred Quinn[c], and Christine M. Szymanski[a,b#]

[a]Department of Microbiology, University of Georgia

[b]Complex Carbohydrate Research Center, University of Georgia

[c]Department of Infectious Diseases, University of Georgia

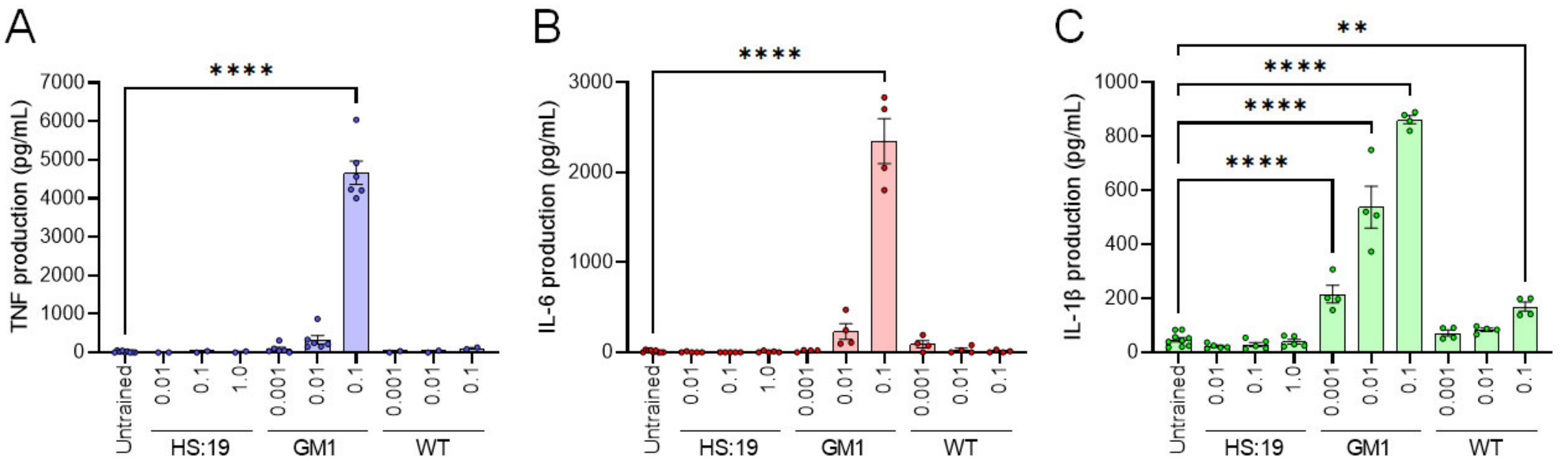

Supplementary Figure 1. Release of cytokines TNF (A), IL-6 (B) and IL-1β (C) after training macrophages with *C. jejuni* HS:19 (HS:19), *E. coli* GM1 (GM1) or *E. coli* WT (WT) at differing multiplicities of infection (MOI) without subsequent challenge. Error bars represent standard error of the mean.
