# Supplementary material for: Low-Dose Exposure to Ganglioside-Mimicking Bacteria Tolerizes Human Macrophages to Guillain-Barré Syndrome-Associated Antigens
Source: mBio. 2022 Feb 1;13(1):e03852-21. doi: 10.1128/mbio.03852-21 (PMC8805021; doi:10.1128/mbio.03852-21)
Supplement: FIG S3 [file mbio.03852-21-sf003.pdf]

Supplemental material – Figure 3

Low-dose exposure to ganglioside-mimicking bacteria tolerizes human macrophages to Guillain-Barré Syndrome-associated antigens

Robert T. Patry[a,b*], Lauren Essler[c], Silke Andresen[a,b], Fred Quinn[c], and Christine M. Szymanski[a,b#]

[a]Department of Microbiology, University of Georgia

[b]Complex Carbohydrate Research Center, University of Georgia

[c]Department of Infectious Diseases, University of Georgia

Methods

Viability staining in trained THP-1 macrophage-like cells

THP-1 monocytes were seeded into 8-well chamber slides at $1.5x10^5$ cells/slide and differentiated as previously described with training ligands. After challenging for 24 hours with *C. jejuni* HS:19, cells were washed twice with PBS and stained with Calcein AM (Invitrogen) for 30 minutes according to the manufacturer's instructions to visualize live cells. The cells were then washed, resuspended in fresh PBS with propidium iodide (ThermoFisher) and imaged immediately using a Zeiss Axiovert 200M microscope at 100x total magnification.

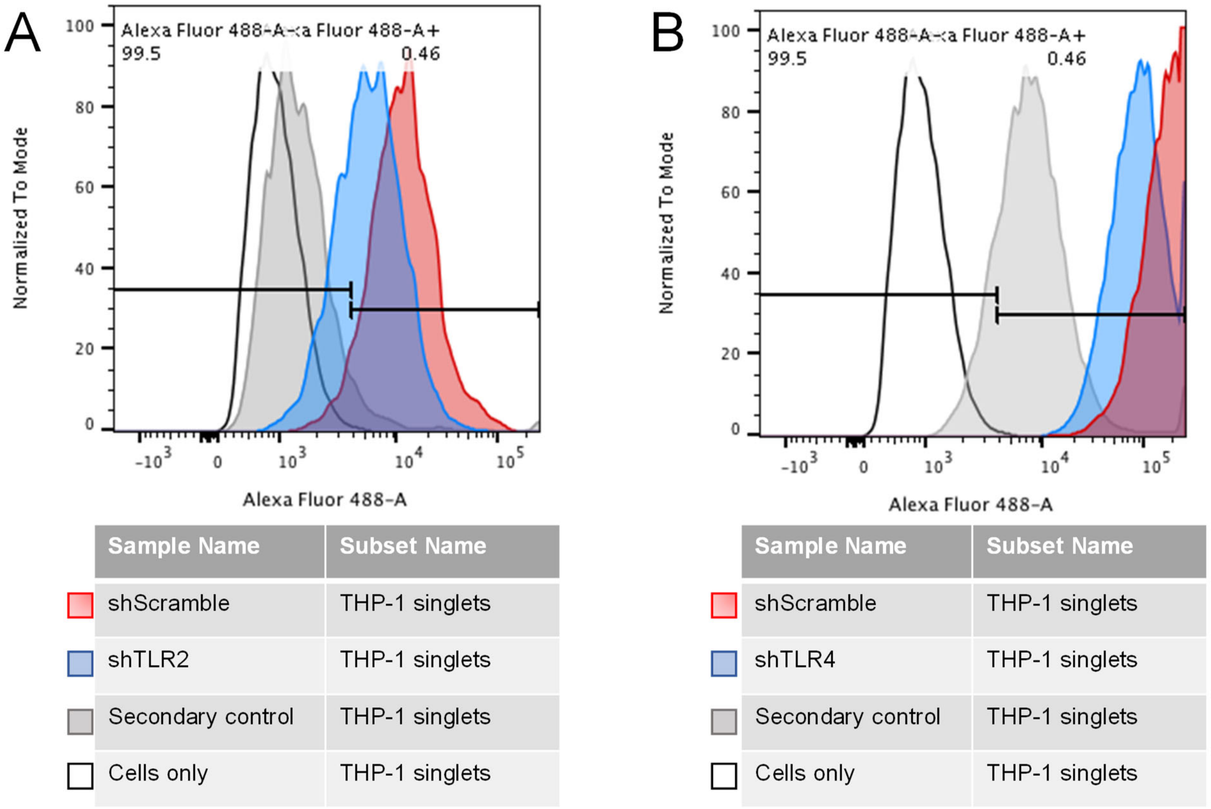

Supplementary Figure 3. Flow cytometry data from surface staining THP-1 cells using TLR2 (A) and TLR4 (B) antibodies in scramble control and the respective TLR knockdown cell lines. TLR expression in scramble control (red) and TLR knockdown cell lines (blue) was compared to unstained (white) and secondary antibody stained (grey) THP-1 cells.
