# Supplementary material for: Low-Dose Exposure to Ganglioside-Mimicking Bacteria Tolerizes Human Macrophages to Guillain-Barré Syndrome-Associated Antigens
Source: mBio. 2022 Feb 1;13(1):e03852-21. doi: 10.1128/mbio.03852-21 (PMC8805021; doi:10.1128/mbio.03852-21)
Supplement: TABLE S1 [file mbio.03852-21-st001.pdf]

Supplemental material – Table 1

Low-dose exposure to ganglioside-mimicking bacteria tolerizes human macrophages to Guillain-Barré Syndrome-associated antigens

Robert T. Patry[a,b*], Lauren Essler[c], Silke Andresen[a,b], Fred Quinn[c], and Christine M. Szymanski[a,b#]

[a]Department of Microbiology, University of Georgia

[b]Complex Carbohydrate Research Center, University of Georgia

[c]Department of Infectious Diseases, University of Georgia

Table S1. Information about infant fecal isolate samples. Describes information regarding CTB western blot results (CTB), strain isolation location, disease symptoms and other pathogens detected in each individual sample. Case and Ctrl represent infants with symptomatic campylobacteriosis and asymptomatic infants, respectively.

Stock Code	Isolate ID	CTB bound	Stool Consistency	Blood, Pus or Mucous?	Pathogens identified	Location	Type
1 A1	400956	Yes	Opaque watery	Mucus	*C. jejuni*	Kenya	Case
1 A3	203886	No	Thick liquid	None	*C. jejuni, E. coli* (ETEC), *E. coli estA* Pos	Mali	Case
1 A5	102878	No	Opaque watery	None	*C. jejuni, E coli* (ETEC), *E. coli eltB* Pos, *Cryptosporidium*	The Gambia	Case
1 A6	103067	Yes	Opaque watery	None	*C. jejuni,* Rotavirus	The Gambia	Case
1 A7	500680	No	Opaque watery	Mucus	*C. jejuni,* Rotavirus	India	Case
1 B1	500429	No	Formed	Blood, Mucus	*C. jejuni*	India	Case
1 B10	704058	No	Soft	None	*C. jejuni, Giardia*	Pakistan	Ctrl
1 B2	204462	No	Rice water-clear watery	Mucus	*C. jejuni, E coli (ETEC), E. coli eltB* Pos	Mali	Case

1 B3	704064	No	Soft	None	*C. jejuni*	Pakistan	Ctrl
1 B4	703886	Yes	Rice water-clear watery	None	*C. jejuni, Rotavirus*	Pakistan	Case
1 B5	704012	No	Soft	None	*Aeromonas, C. jejuni, Salmonella* non-typhi*, E. coli* (EAEC)*, E. coli aatA* Pos	Pakistan	Ctrl
1 B6	703934	No	Thick liquid	Blood, Mucus	*Aeromonas, C. jejuni, Rotavirus, Adenovirus, Adenovirus* 40/41, *Norovirus* GII	Pakistan	Case
1 B7	704017	No	Soft	None	*C. jejuni, Sapovirus*	Pakistan	Ctrl
1 B8	704138	No	Soft	Mucus	*C. jejuni, Cryptosporidium, Adenovirus*	Pakistan	Ctrl
1 B9	704137	No	Soft	None	*C. jejuni, E. coli* (EAEC)*, E. coli aatA* Pos, *Cryptosporidium, Norovirus* GII	Pakistan	Ctrl
1 C10	700544	No	Rice water-clear watery	None	*Aeromonas, C. jejuni*	Pakistan	Case
1 C2	702567	Yes	Soft	Mucus	*C. jejuni*	Pakistan	Ctrl
1 C3	703207	Yes	Rice water-clear watery	None	*C. jejuni, Sapovirus*	Pakistan	Case
1 C4	703312	No	Thick liquid	None	*C. jejuni, Sapovirus*	Pakistan	Case
1 C5	702621	No	Thick liquid	None	*C. jejuni, E coli* (EAEC)*, E. coli aatA* Pos, *Norovirus* GI	Pakistan	Ctrl
1 C6	702647	No	Thick liquid	Mucus	*C. jejuni, E. coli* (EAEC)*, E. coli aatA* Pos	Pakistan	Ctrl
1 C7	702893	No	Opaque watery	None	*C. jejuni, E. coli* (EAEC)*, E. coli aatA* Pos, *E. coli aaiC* Pos, *Norovirus* GII, *Sapovirus*	Pakistan	Case
1 C8	700541	No	Opaque watery	None	*Aeromonas, C. jejuni, V. cholerae, Rotavirus*	Pakistan	Case
1 C9	700006	No	Rice water-clear watery	None	*Aeromonas, C. jejuni, E. coli* (ETEC)*, E. coli estA* Pos, *E. coli eltB* Pos	Pakistan	Case
1 D1	700690	No	Opaque watery	None	*C. jejuni, V. cholerae, E. coli* (tEPEC)	Pakistan	Case
1 D10	710352	No	Soft	None	*C. jejuni, E. coli* (EPECeae)	Pakistan	Ctrl
1 D2	702296	No	Thick liquid	None	*C. jejuni, E. coli* (aEPEC)	Pakistan	Case

1 D3 and J1	702519	No	Opaque watery	None	*C. jejuni, Norovirus* GII	Pakistan	Case
1 D4 and J3	703533	No	Rice water-clear watery	None	*C. jejuni, E coli* (tEPEC)	Pakistan	Case
1 D5	720383	No	Soft	None	*C. jejuni, E. coli* (EPEC, *eae*)	Pakistan	Ctrl
1 D6	720420	Yes	Opaque watery	None	*C. jejuni, E. coli* (EPEC, *eae*)	Pakistan	Case
1 D7	720390	Yes	Soft	None	*C. jejuni, E. coli* (EPEC, *eae, aaiC*)	Pakistan	Ctrl
1 D8	710444	No	Soft	None	*C. jejuni, E. coli (EPEC, eae)*	Pakistan	Ctrl
1 D9	710136	No	Soft	None	*C. jejuni, E coli* (EPEC, *eae, aatA, aaiC*)	Pakistan	Ctrl
1 E1	710351	Yes	Opaque watery	None	*C. jejuni, Giardia lamblia, Adenovirus, E. coli* (EPEC, *eae, aatA*)	Pakistan	Case
1 E10	720437	No	Thick liquid	None	*C. jejuni, Giardia lamblia, Rotavirus, Norovirus* GII, *S. boydii, E. coli* (EPEC, *eae*)	Pakistan	Case
1 E2	720296	No	Opaque watery	None	*C. jejuni, Astrovirus, B.fragilis, E. coli* (EPEC, *eae, aatA*)	Pakistan	Case
1 E3	720284	No	Soft	None	*C. jejuni, Adenovirus, B.fragilis, E coli* (EPEC, *eae*)	Pakistan	Ctrl
1 E4	710121	No	Rice water-clear watery	None	*C. jejuni, E. coli* (EPEC, *eae*)	Pakistan	Case
1 E5	710236	No	Opaque watery	None	*C. jejuni, V. cholerae, Vibrio* 132, *Astrovirus, E. coli* (EPEC, *eae, aaiC*)	Pakistan	Case
1 E6	720032	No	Opaque watery	None	*C. jejuni, Helicobacter pylori, E. coli* (EPEC, *eae*)	Pakistan	Case
1 E7	610396	Yes				Bangladesh	Case
1 E8	620425	No	Formed	None	*C. jejuni, Clostridium difficile* (GDH, Ag), *E. coli* (EPEC, *eae*)	Bangladesh	Ctrl
1 E9	720607	No	Thick liquid	None	*C. jejuni, E. coli* (EPEC, *eae, aaiC*)	Pakistan	Ctrl
1 F1	320435	Yes	Formed	None	*C. jejuni, Giardia lamblia, E coli* (EPEC, *eae, bfpA*)	Mozambique	Ctrl
1 F10	603060	Yes	Formed	None	*Aeromonas, C. jejuni, Salmonella* non-typhi, *E. coli* (ETEC), *E. coli eltB* Pos, *Giardia*	Bangladesh	Ctrl

1 F2	710293	No	Soft	None	*C. jejuni, Helicobacter pylori, Norovirus* GII, *E coli* (EPEC*, eae, bfpA*)	Pakistan	Ctrl
1 F3	720075	No	Opaque watery	Mucus	*C. jejuni, E. coli* (EPEC, *eae*)	Pakistan	Case
1 F4	720005	No	Thick liquid	Pus, Mucus	*C. jejuni, E. coli* (EPEC, *eae, aatA*)	Pakistan	Case
1 F5	603771	No	Formed	None	*Aeromonas, C. jejuni*	Bangladesh	Ctrl
1 F6	600099	No	Opaque watery	None	*C. jejuni, E. coli* (EAEC), *E. coli aaiC* Pos, *Cryptosporidium, Rotavirus*	Bangladesh	Case
1 F7	600021	No	Soft	Blood, Mucus	*C. jejuni*	Bangladesh	Case
1 F8	604419	No	Soft	Blood, Mucus	*Aeromonas, C. jejuni, Salmonella* non-typhi, *E. coli* (tEPEC), *E. coli bfpA* Pos	Bangladesh	Case
1 F9	603180	No	Thick liquid	Mucus	*Aeromonas, C. jejuni, Rotavirus*	Bangladesh	Case
1 G1	710087	No	Soft	None	*C. jejuni, Giardia lamblia, Helicobacter pylori, Sapovirus, E. coli (*EPEC*, eae, aaiC*)	Pakistan	Ctrl
1 G10	403459	No	Thick liquid	Mucus	*C. jejuni*	Kenya	Case
1 G2	720255	No	Thick liquid	None	*C. jejuni, E. coli* (EPEC, *eae, aaiC*)	Pakistan	Case
1 G3	720147	No	Soft	None	*C. jejuni, Clostridium difficile* (GDH *Ag*) *E. coli* (EPEC*, eae*)	Pakistan	Ctrl
1 G4	600978	No	Soft	Blood, Mucus	*Aeromonas, C. jejuni, E. coli* (EAEC), *E. coli aatA* Pos, *Rotavirus*	Bangladesh	Case
1 G5	600883	No	Thick liquid	Mucus	*C. jejuni*	Bangladesh	Case
1 G6	601037	Yes	Thick liquid	Mucus	*C. jejuni*	Bangladesh	Case
1 G7	704231	No	Soft	None	*C. jejuni, Cryptosporidium*	Pakistan	Case
1 G8	400738	No	Thick liquid	Mucus	*C. jejuni, E. coli* (tEPEC), *E. coli bfpA* Pos, *E. coli eae* Pos, *Adenovirus*	Kenya	Case
1 G9	400526	No	Opaque watery	Mucus	*C. jejuni, E. coli* (aEPEC), *E coli eae* Pos	Kenya	Case
1 H1	403205	No	Opaque watery	None	*C. jejuni, Cryptosporidium*	Kenya	Case

1 H10	703664	No	Opaque watery	None	*C. jejuni, E. coli* (ETEC), *E. coli estA* Pos, *E. coli eltB* Pos	Pakistan	Case
1 H2	401022	No	Formed	None	*C. jejuni, E. coli* (EAEC), *E. coli aaiC* Pos	Kenya	Ctrl
1 H3	402870	No	Opaque watery	None	*C. jejuni, E. coli* (aEPEC)	Kenya	Case
1 H4	401051	No	Soft	None	*C. jejuni*	Kenya	Ctrl
1 H5	403385	No	Opaque watery	Mucus	*C. jejuni*	Kenya	Case
1 H6	320256	No	Formed	None	*C. jejuni, E coli* (EPEC*, eae*)	Mozambique	Ctrl
1 H7	403257	No	Soft	None	*C. jejuni, Entamoeba*	Kenya	Ctrl
1 H8	703672	No	Soft	None	*C. jejuni, E. coli* (EAEC), *E. coli aatA* Pos, *E. coli aaiC* Pos, *Norovirus* GI	Pakistan	Ctrl
1 H9	703637	Yes	Soft	None	*C. jejuni, Salmonella non-typhi, E. coli* (EAEC), *E. coli* (tEPEC), *E. coli* (aEPEC), *E. coli aaiC* Pos, *E. coli eae* Pos, *Astrovirus*	Pakistan	Case
1 I1	703882	No	Soft	None	*C. jejuni, Norovirus* GII	Pakistan	Ctrl
1 I10	721262	No	Opaque watery	None	*C. jejuni, Rotavirus, Norovirus* GII, *E. coli* (EPEC *eae, bfpA*)	Pakistan	Case
1 I2	703779	No	Rice water-clear watery	None	*C. jejuni, Cryptosporidium, Rotavirus*	Pakistan	Case
1 I3	703871	Yes	Soft	Mucus	*Aeromonas, C. jejuni, Salmonella* non-typhi, *Norovirus* GI	Pakistan	Ctrl
1 I4	720906	No	Soft	None	*Aeromonas, C. jejuni, Sapovirus, E. coli* (EPEC *eae, eltB*)	Pakistan	Ctrl
1 I6	710603	No	Rice water-clear watery	None	*C. jejuni, E. coli* (EPEC, *eae, aaiC*)	Pakistan	Case
1 I7	720839	No	Soft	Mucus	*C. jejuni, S. flexneri* 4a, *E. coli* (EPEC, *eae*)	Pakistan	Case
1 I8	110498	Yes	Opaque watery	Mucus	*C. jejuni*	The Gambia	Case
1 I9	721314	Yes	Soft	None	*C. jejuni, Clostridium difficile* (GDH Ag*) E. coli* (EPEC*, eae, aatA, aaiC*)	Pakistan	Case

1 J10	520188	No	Formed	Pus, Mucus	*C. jejuni, Clostridium difficile* GDH Ag, *E coli* (EPEC*, eae*)	India	Ctrl
1 J2	120617	Yes	Thick liquid	None	*C. jejuni, Clostridium difficile* GDH Ag, *B.fragilis, E coli* (EPEC, *eae, aatA*)	The Gambia	Ctrl
1 J4	721311	No	Soft	None	*C. jejuni, E coli* (EPEC*, eae, aatA*)	Pakistan	Ctrl
1 J5	103169	Yes	Thick liquid	None	*C. jejuni*	The Gambia	Ctrl
1 J6	503818	No	Opaque watery	Pus, Mucus	*C. jejuni, E. coli* (EAEC)*, E. coli* (tEPEC)*, E. coli bfpA* Pos, *E coli aaiC* Pos, *E coli eae* Pos, *Giardia, Cryptosporidium*	India	Case
1 J7	504021	Yes	Opaque watery	None	*C. jejuni*	India	Case
1 J8	504343	Yes	Soft	Mucus	*C. jejuni*	India	Ctrl
1 J9	520016	Yes	Soft	Pus, Mucus	*C. jejuni, E coli* (EPEC*, eae, aatA*)	India	Case
2 A1	504076	No	Thick liquid	Mucus	*C. jejuni, Cryptosporidium*	India	Case
2 A10	510831	No	Thick liquid	Pus, Mucus	*C. jejuni, Adenovirus, Adenovirus* 40/41, *E. coli* (EPEC, *eae*)	India	Case
2 A2	504764	No	Formed	None	*C. jejuni, E coli* (EAEC)*, E coli aaiC* Pos, *Giardia, Cryptosporidium*	India	Ctrl
2 A3	504611	No	Opaque watery	Mucus	*C. jejuni*	India	Case
2 A4	504977	No	Soft	Pus, Mucus	*C. jejuni, E. coli* (EAEC), *E. coli aatA* Pos, *E. coli aaiC* Pos	India	Ctrl
2 A5	505394	Yes	Soft	Mucus	*C. jejuni*	India	Ctrl
2 A6	504196	Yes	Formed	None	*C. jejuni, E. coli* (EAEC), *E. coli aatA* Pos	India	Ctrl
2 A7	503610	No	Soft	Mucus	*C. jejuni, Cryptosporidium, Rotavirus*	India	Case
2 A8	503674	No	Opaque watery	None	*C. jejuni, Cryptosporidium, Rotavirus*	India	Case
2 A9	504730	No	Soft	None	*C. jejuni, Adenovirus*	India	Ctrl
2 B1	510942	Yes	Soft	Pus, Mucus	*C. jejuni, Salmonella* non-typhi, *Clostridium difficile*	India	Ctrl

					GDH Ag, *E. coli* (EPEC*eae*)		
2 B10	703833	Yes	Soft	None	*Aeromonas, C. jejuni*	Pakistan	Case
2 B2	511171	No	Soft	Mucus	*C. jejuni, Clostridium difficile* GDH Ag, *E. coli* (EPEC, *eae*)	India	Ctrl
2 B3	520190	No	Soft	Pus, Mucus	*C. jejuni, E. coli (EPEC, eae*)	India	Ctrl
2 B4	520430	No	Thick liquid	Pus, Mucus	*C. jejuni, E. coli* (EPEC*, eae*)	India	Case
2 B5	521136	Yes	Soft	Mucus	*C. jejuni, Clostridium difficile* GDH Ag, *E. coli* (EPEC*, eae, estA*)	India	Ctrl
2 B6	521162	No	Soft	Mucus	*C. jejuni, E coli* (EPEC*, eae*)	India	Ctrl
2 B7	703493	No	Soft	None	*C. jejuni, Cryptosporidium*	Pakistan	Ctrl
2 B8	703438	No	Thick liquid	Mucus	*C. jejuni*	Pakistan	Case
2 B9	703777	No	Opaque watery	None	*C. jejuni, Cryptosporidium*	Pakistan	Case
2 C1	710652	Yes	Opaque watery	None	*Aeromonas, C. jejuni, Salmonella* non-typhi, *V. cholerae, Vibrio* 13V inaba ogawa, *E. coli* (EPEC, *eae*)	Pakistan	Case
2 C2	720972	No	Thick liquid	None	*C. jejuni, Cryptosporidium spp., Norovirus* GI, *E. coli* (EPEC*, eae, bfpA*)	Pakistan	Case
2 C3	721225	No	Soft	Mucus	*C. jejuni, Norovirus* GII, *E. coli* (EPEC, *eae, aatA, aaiC*)	Pakistan	Ctrl
2 C4	721253	No	Soft	None	*C. jejuni, Clostridium difficile* GDH Ag, *E. coli* (EPEC*, eae*)	Pakistan	Case
2 C5	721319	No	Soft	None	*C. jejuni, Giardia lamblia, E. coli* (EPEC, *eae, aatA*)	Pakistan	Ctrl
3 1	201666	No				Mali	Ctrl
3 5	203084	No	Soft	None	*Campylobacter* nonspec, *E. coli* (EAEC), *E coli aatA* Pos, *E coli aaiC* Pos, *Giardia, Sapovirus*	Mali	Ctrl
3 6	203106	No	Thick liquid	Mucus	*Campylobacter* nonspec	Mali	Case
3 8	203460	No	Soft	None	*Campylobacter* nonspec	Mali	Ctrl

3 9	203524	No	Thick liquid	None	*Campylobacter* nonspec, *E. coli* (EAEC)*, E. coli aaiC Pos*	Mali	Case
3 10	204409	Yes	Opaque watery	None	*C. jejuni, Rotavirus*	Mali	Ctrl
3 11	310356	No				Mozambique	Case
3 12	310457	Yes	Formed	None	*C. jejuni, Entamoeba histolytica, E. coli* (EPEC, *eae*)	Mozambique	Ctrl
3 13	320061	Yes	Opaque watery	None	*C. jejuni, E. coli* (EPEC, *eae, bfpA*)	Mozambique	Case
3 14	320644	No	Opaque watery	None	*C. jejuni, Rotavirus, E. coli* (EPEC, *eae*)	Mozambique	Case
3 15	500963	No	Formed	None	*C. jejuni*	India	Ctrl
3 16	503093	No	Opaque watery	None	*C. jejuni*	India	Case
3 17	503603	No	Opaque watery	None	*C. jejuni, E. coli* (aEPEC)*, E. coli eae* Pos, *Cryptosporidium*	India	Case
3 18	504361	Yes	Soft	None	*C. jejuni*	India	Ctrl
3 19	600035	Yes	Soft	Mucus	*C. jejuni, E. coli* (ETEC), *E. coli eltB* Pos, *Cryptosporidium*	Bangladesh	Case
3 20	703317	No	Soft	None	*Aeromonas, C. jejuni, E. coli* (aEPEC)	Pakistan	Ctrl
3 21	710670	No	Soft	None	*C. jejuni, Giardia lamblia, Norovirus* GII, *E. coli* (*EPEC, eae, aatA, aaiC*)	Pakistan	Ctrl
3 22	710796	No	Opaque watery	None	*C. jejuni, Cryptosporidium spp., S. flexneri* 3a, *E. coli* (EPEC*, eae, bfpA,* Stx2, Stx1)	Pakistan	Case
3 23	710963	No	Soft	None		Pakistan	Ctrl
3 41	100801	Yes	Thick liquid	None	*C. coli, E. coli* (EAEC)*, E. coli aaiC* Pos	The Gambia	Ctrl
3 42	121307	No	Opaque watery	Mucus	*C. coli, E. coli* (EPEC*, eae*)	The Gambia	Case
3 43	202388	No	Opaque watery	Mucus	*Campylobacter* nonspec	Mali	Case
3 44	204304	No	Thick liquid	Mucus	*Campylobacter* nonspec, *Giardia*	Mali	Case
3 45	400679	No	Thick liquid	Mucus	*C. coli, E. coli* (aEPEC), *E. coli eae* Pos	Kenya	Case
3 46	400879	No	Thick liquid	Blood, Pus, Mucus	*C. coli, Cryptosporidium*	Kenya	Case

3 47	403965	No	Thick liquid	Mucus	*C. coli, Rotavirus*	Kenya	Case
3 48	500573	Yes	Soft	None	*C. coli, E. coli* (EAEC), *E. coli aatA* Pos	India	Ctrl
3 49 & 50	403362	Yes	Opaque watery	Mucus	*C. coli, E. coli* (tEPEC), *E. coli bfpA* Pos, *E. coli eae* Pos, *Norovirus* GI	Kenya	Case
3 51	505506	No	Soft	Mucus	*C. coli, E. coli* (ETEC), *E. coli estA* Pos	India	Ctrl
3 52	505965	No	Soft	Pus, Mucus	*C. coli, V. cholerae*	India	Ctrl
3 53	510333	No	Soft	None	*C. coli, Clostridium difficile* GDH Ag, *E. coli* (EPEC, *eae*)	India	Ctrl
3 54 & 55	601035	No	Formed	None	*C. coli, E. coli* (ETEC), *E. coli eltB* Pos	Bangladesh	Ctrl
3 56	602330	Yes	Formed	None	*Aeromonas, C. coli*	Bangladesh	Ctrl
3 57	602497	No	Thick liquid	Blood, Mucus	*Aeromonas, C. coli, E. coli* (ETEC), *E. coli eltB* Pos	Bangladesh	Case
3 58	602723	No	Formed	None	*C. coli, V. cholerae, E. coli* (EAEC), *E. coli aaiC* Pos	Bangladesh	Ctrl
3 59	603567	No	Formed	None	*C. coli, Astrovirus*	Bangladesh	Ctrl
3 60	610001	No	Thick liquid	Mucus	*Aeromonas, C. coli, Giardia lamblia, Clostridium difficile* GDH Ag, *E. coli* (EPEC, *eae*)	Bangladesh	Case
3 61	610647	No	Thick liquid	Mucus	*C. coli, V. cholerae, Vibrio* non, *S. sonnei, E coli* (EPEC, *eae*)	Bangladesh	Case
